# The role of mitochondrial transfer via tunneling nanotubes in the central nervous system: A review

**DOI:** 10.1097/MD.0000000000037352

**Published:** 2024-03-01

**Authors:** Ye Chen, Dongqiong Xiao, Xihong Li

**Affiliations:** a Department of Emergency Medicine, West China Second University Hospital, Sichuan University, Chengdu, China; b Key Laboratory of Birth Defects and Related Diseases of Women and Children (Sichuan University), Ministry of Education, Chengdu, China.

**Keywords:** central nervous system, mitochondrial transfer, TNFAIP2, TNTs

## Abstract

Tumour necrosis factor alpha-induced protein 2 (TNFAIP2) is a gene induced by tumor necrosis factor in endothelial cells. TNFAIP2 has important functions in physiological and pathological processes, including cell proliferation, adhesion, migration, angiogenesis, inflammation, tunneling nanotube (TNT) formation and tumorigenesis. Moreover, TNFAIP2 is the key factor in the formation of TNTs. TNTs are related to signal transduction between different cell types and are considered a novel means of cell-to-cell communication. Mesenchymal stem cells (MSCs) are pluripotent cells that exhibit self-renewal, multidirectional differentiation, paracrine function and immune-regulating ability. MSCs can transfer mitochondria through TNTs to improve the functions of target cells. This review revealed that TNFAIP2 promotes the formation of TNTs and that MSCs rely on TNTs for mitochondrial transfer to ameliorate cell dysfunction.

## 1. Introduction

**Figure 1.**
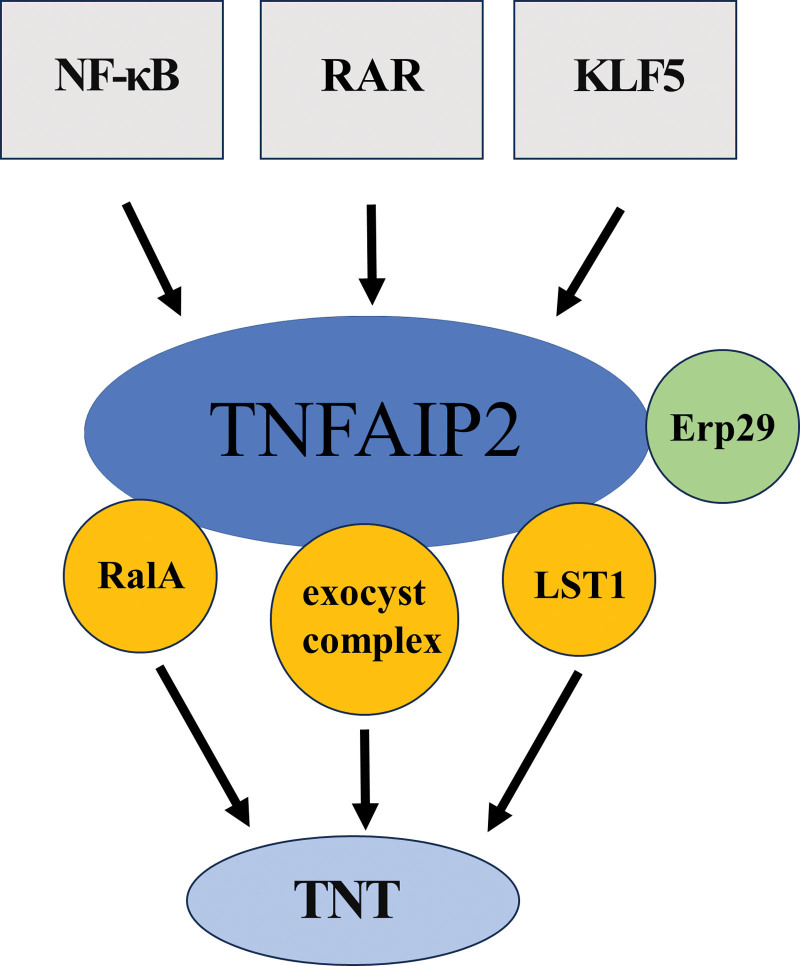
The function, regulatory mechanism and interacting proteins of TNFAIP2. TNFAIP2 = tumor necrosis factor alpha-induced protein 2.

Tumour necrosis factor alpha (TNF-α) is a cytokine produced by macrophage/monocyte activation. TNF-α is involved in a variety of processes, including cell division and differentiation, apoptosis, lipid metabolism, and inflammation.^[1]^ TNF-α-induced proteins (TNFAIPs) are a family of proteins induced by TNF-α that are involved in a variety of biological processes. A member of the TNFAIP family is tumor necrosis factor alpha-induced protein 2 (TNFAIP2), often referred to as B94 or M-Sec. This gene has 14 exons and is found on human chromosome 14q32.32. The genomic DNA comprises 13.45 kb and encodes a 72.6 kDa protein composed of 654 amino acids.^[2]^ It is mainly distributed outside the plasma membrane and was initially thought to be a new gene induced by TNF-α in human endothelial cells. TNFAIP2 is found mainly on immune cells, such as monocytes, endothelial cells, dendritic cells, intestinal M cells and macrophages.^[3]^ Moreover, TNFAIP2 plays multiple roles in organ formation and development. It can regulate inflammation and angiogenesis through the nuclear factor-κB (NF-κB) signaling pathway, the retinoic acid signaling pathway and the Krüppel-like factor 5 signaling pathway. Moreover, it plays important roles in cell differentiation, cell proliferation, material transport and tunneling nanotube (TNT) formation^[4]^ (Fig. 1).

## 2. Introduction of TNTs

### 2.1. Definition and function of TNTs

In recent years, TNTs have attracted increasing amounts of attention as a novel method of intercellular communication. TNTs are defined by the following criteria^[5]^: a length of thin (20–700 nm diameter) or crude (>700 nm diameter) membranous processes that hover over the cell matrix, connecting two or more cells; the existence of a cytoskeleton rich in F-actin; transport cargo from donor cells to recipient cells; and have open cell–cell contact sites at both ends that allow cytoplasmic continuity. Notably, the ability to transport goods is an important difference between TNTs and other closed membranous processes.

TNTs have been found in various cell lines, including primary rat neurons, astrocytes, and immune cells, since their discovery in 2004 in cultured rat pheochromocytoma PC12 cells.^[6,7]^ TNTs are transient fibrous membranes connecting cells that are composed of cell membranes, actin, myosin and tubulin. Moreover, TNTs are the protuberant part of the plasma membrane and enable the plasma membranes of distal cells to be directly physically connected for functional continuity.^[8]^ TNTs can also be formed between different types of cells for intercellular communication.^[9]^

TNTs consist of tubular cytoplasmic ducts of various lengths and membrane closures that mediate the transmission of various signaling molecules and cellular components, such as organelles (vesicles, lysosomes, mitochondria, autophagosomes, etc.), macromolecules (such as nucleic acids and proteins), and tiny molecules (such as calcium ions).^[10–12]^ In addition, TNTs can mediate intercellular virus transmission and participate in cell damage repair, immune response activation and cellular metabolic reprogramming.^[13]^

### 2.2. The formation process of TNTs

To date, 2 different mechanisms for the formation of TNTs have been proposed. The first is sometimes referred to as the “cellular dislocation mechanism” and describes 2 cells that are adjacent to one another temporarily fusing and maintaining a membrane line during separation, followed by the creation of TNTs.^[14]^ The cells that have been observed to undergo this process include macrophages, NK cells, T cells, and other kinds of immune cells.^[15]^ The second mechanism, referred to as actin-driven processes, involves the formation of a pseudopod-like process from one cell to another; this process extends and eventually fuses with neighboring cells to form TNTs.^[16]^ These 2 processes are not necessarily mutually exclusive during TNT formation, and they can even occur at the same time. Real-time imaging has shown that cell dislocation is the main mechanism of TNT formation in many cell types.^[6,17]^

TNTs are membrane protuberances supported by the actin cytoskeleton, but TNTs exhibit different phenotypes. The difference lies in their cytoskeletal composition and functional properties.^[18]^ As mentioned earlier, TNTs can be divided into 2 types: fine TNTs (diameter < 700 nm) containing only F-actin and crude TNTs (diameter > 700 nm) containing both F-actin and microtubules. While thin TNTs include only F-actin, which indicates unidirectional transport, coarse TNTs appear to be associated with the bidirectional transfer of cellular components, including organelles.^[19]^

TNTs are extremely sensitive to external stimuli, as demonstrated by earlier investigations investigating healthy and pathological conditions.^[20]^ In the presence of oxidative stress, inflammation, radiation or trauma and apoptosis, the formation of TNTs greatly increases.^[21]^ In addition, exposure to adverse environmental pressures can not only increase the formation of TNTs but also accelerate the transport of various substances to improve cell function.^[22]^ These findings suggest that TNTs can help cells adapt to adverse environmental conditions. The ability of cells to connect and share resources through TNTs represents an effective rescue mechanism that provides a new way for damaged cells to survive.^[23]^

### 2.3. TNFAIP2 and TNTs

TNTs are the most recently discovered type of intercellular signal transduction technique; they facilitate the interchange of cellular components and signal transduction molecules and are crucial for cell-to-cell communication. Because there are no distinct TNT markers, previous studies have relied on morphological analysis, so it is difficult to accurately identify TNTs and TNT-like structures. TNFAIP2 is a marker of TNT formation and is the central factor in TNT formation.^[24]^ There are many examples of TNFAIP2 promoting TNT formation. For example, lipopolysaccharides can induce an acute inflammatory response and increase the expression of TNFAIP2.^[25]^ Rat hippocampal astrocytes were treated with hydrogen peroxide, which increased the expression of TNFAIP2 and the production of TNTs.^[26]^ Infection with human T-cell lymphoma virus 1 induces the expression of TNFAIP2 mRNA in T cells and increases the formation of TNTs.^[27]^ Human immunodeficiency virus-1 induces TNFAIP2 expression and promotes the formation of TNTs in T cells, thus enhancing the intercellular transmission of HIV-1.^[28]^ Podocytes in the kidney can express TNFAIP2. Exposure to serum deprivation or adriamycin induces an increase in TNFAIP2 expression and TNT formation.^[29]^ Overexpression of TNFAIP2 promoted the formation of TNTs, while inhibition of TNFAIP2 expression prevented the formation of TNTs. Moreover, TNFAIP2 is necessary for the formation of TNTs.^[30]^

The identification of TNFAIP2 as a marker of TNTs and as a promoter of TNT formation will aid in the elucidation of the mechanisms involved in the development of these structures. The exact mechanism through which TNFAIP2 promotes TNT formation is discussed below.

#### 2.3.1. TNFAIP2 promotes the formation of TNTs by interacting with RalA and the external capsule complex

The Ras-related protein Ral belongs to the small GTP enzyme superfamily. The Ral family is a class of low-molecular-weight proteins with 2 forms: inactive Ral-GDP and active Ral-GTP.^[31]^ Ral has 2 homologous isomers, RalA and RalB. RalA is a multifunctional small GTPase that participates in cell material transport, cell proliferation, cell migration, apoptosis, carcinogenesis and actin cytoskeleton remodeling.^[32]^ Studies have shown that TNFAIP2 is a key molecule in the formation of TNTs. The synthesis of functional TNTs may be induced by the cooperative action of TNFAIP2, RalA, and the exocyst complex.^[33]^

The formation of TNTs induced by TNFAIP2 is accompanied by actin cytoskeleton remodeling. Ral participates in the regulation of actin cytoskeleton remodeling through a variety of mechanisms.^[34]^ First, Ral-GTP interacts directly with the Silk protein, which is subsequently drawn to the plasma membrane, where it stimulates the growth of filamentous pseudopodia by binding to the carboxyl terminal domain of RalA. Second, under the influence of TNFAIP2, Ral-GTP binds to Ral binding protein 1, which is involved in the regulation of actin remodeling and in the creation of filamentous pseudopodia. This increases the expansion of the membrane process. Third, by interacting with the exocyst complex, RalA-GTP promotes the development of filamentous pseudopodia.^[35]^ TNFAIP2 triggers F-actin polymerization through cooperation with RalA and extracellular complexes. In addition, TNFAIP2 induces membrane processes to extend outwards from the plasma membrane, and some of these membrane processes are connected to neighboring cells, thus forming TNTs. In other words, TNFAIP2-induced TNT formation is related to F-actin, which plays a key role in TNT formation, as it has been found that inhibition of actin polymerization can prevent the formation of TNTs.^[36]^ These findings imply that the exocyst complex may facilitate actin cytoskeleton remodeling and membrane replenishment, which are required for TNFAIP2-induced membrane protrusion. In addition, RalA and the exocyst complex interact with the single-channel membrane protein known as leukocyte-specific transcript 1 protein (LST1). LST1 expressed in the plasma membrane may function as a scaffold to encourage the interaction of molecules that regulate the production of TNTs.^[37]^

TNFAIP2, together with RalA, LST1 and the exocyst complex, initiates the formation and extension of TNTs by regulating the polymerization of actin and the transport of the membrane sac to the protruding area of the cell surface to produce TNTs.^[38]^

#### 2.3.2. The various functions of the N-terminal and C-terminal domains of TNFAIP2 in the deformation of the TNT plasma membrane

The plasma membrane is where TNFAIP2 localizes, which is mediated via its N-terminus. While the positively charged C-terminal surface interacts with active RalA to promote the extension of TNTs, the N-terminus may act as a switch to initiate the deformation of the membrane, which is also necessary for its function. The process of TNT formation induced by TNFAIP2 is as follows^[33]^: In the initial stage of TNT formation, the N-terminal polybase region of TNFAIP2 directly binds phosphatidylinositol (4,5)-bisphosphate ([PI (4,5) P2]) to the plasma membrane. Subsequently, membrane-bound TNFAIP2 recruits active RalA through its positively charged C-terminal surface, causes membrane deformation, and cooperates with the exocyst complex and LST1 to promote the formation of membrane extensions.^[39,40]^ From this point, a short TNT slowly spreads outwards and gradually touches the plasma membrane of neighboring cells to produce a functioning TNT.

#### 2.3.3. ERp29 regulates TNT formation by stabilizing TNFAIP2

In view of the extensive functions of TNTs across a variety of cell lines and organisms, additional cellular proteins may interact with TNFAIP2 to help form TNTs. Endoplasmic reticulum protein 29, a resident protein of the ER with a molecular weight of 29 kDa, has been the subject of extensive research as a novel chaperone of the protein-disulfide isomerase family. To maintain stable function, it binds to a variety of protein substrates.^[41]^

ERp29 expression has a significant effect on the protein expression level of TNFAIP2 but has no significant effect on its mRNA level, which indicates that ERp29 acts on TNFAIP2 as a protein chaperone.^[42]^ Deletion of the ERp29 protein leads to a decrease in the cellular level of TNFAIP2 and a significant decrease in the number of TNTs.^[43]^ The C-terminal domain of ERp29, which is crucial for this function of the RalA-exocyst complex, has been shown to be unaffected by the deletion of ERp29.^[44]^

The interaction between TNFAIP2 and ERp29 is a prerequisite for its final localization to the plasma membrane and for the initiation of TNT formation.^[43]^ Therefore, ERp29 may be an important link in the cellular mechanism required for TNT formation. The interaction between TNFAIP2 and ERp29 is a prerequisite for the correct localization of TNFAIP2 to the plasma membrane. Without this stabilization effect, TNFAIP2 could not induce the formation of TNTs. An important upstream molecular mechanism is thus revealed here. ERp29 regulates the formation of TNTs by stabilizing TNFAIP2.

#### 2.3.4. The NF-κB signaling pathway is involved in TNFAIP2-mediated formation of TNTs

Reactive oxygen species (ROS) can damage endothelial cells, increase vascular endothelial permeability, cause exudation of plasma proteins, and cause tissue and organ edema and dysfunction. ROS activate NF-κB in corneal epithelial cells and promote the formation of TNTs by upregulating TNFAIP2. SC-514-mediated inhibition of the NF-κB pathway decreases the expression level of TNFAIP2 and the efficiency of TNT formation and blocks the formation of TNTs induced by TNFAIP2.^[45]^ It is hypothesized that TNFAIP2 is primarily responsible for the synthesis of TNTs through the NF-κB signaling pathway. Additionally, TNFα may activate NF-κB in MSCs to increase the expression of TNFAIP2, which promotes the production of TNTs.^[46]^

## 3. TNTs and mitochondrial transfer

### 3.1. Introduction to mitochondrial transfer

Most eukaryotic cells contain mitochondria, which are double-membraned organelles with a high degree of dynamic activity. By regulating calcium signaling, energy production, and cellular metabolism, mitochondria govern intracellular homeostasis.^[47]^ Cellular energy is generated by mitochondria, which efficiently produce ATP and a small amount of the harmful oxidant ROS.^[48]^ Mitochondria play a crucial role in cellular physiology and energy synthesis. Both physiologically and pathologically, intercellular mitochondrial transfer across different cells has been observed in vitro and in vivo.^[49]^ Mitochondrial activity can affect a variety of functions in MSCs, such as differentiation, senescence, immunomodulation, apoptosis, proliferation, migration and chemotaxis.

#### 3.1.1. The pattern of mitochondrial transfer

A novel and intriguing method of intercellular communication is mitochondrial transfer. Most related research has demonstrated that mitochondrial transfer occurs through TNTs, extracellular vesicles, and gap junction channels.^[50]^ The key structure in TNTs is F-actin. F-actin cross-linking ensures the rigidity of the TNT and endows the TNT with outwards growth stability and an appropriate processing length for resisting buckling.^[51]^ Conversely, F-actin cross-linking can allow for the transport of mitochondria along the cytoskeletal structure of TNTs.^[52]^ Interestingly, TNT-mediated mitochondrial transfer can be unidirectional or bidirectional.^[53]^ This finding shows that mitochondrial transport mediated by TNTs is functional and well controlled.

#### 3.1.2. Markers of mitochondrial transfer

Numerous intracellular and extracellular processes in recipient cells, such as hypoxia-induced stress, oxygen and glucose deprivation (OGD), drug-induced oxidative stress, inflammation, and others, frequently cause intercellular mitochondrial transfer.^[54–56]^ Furthermore, to initiate mitochondrial transfer, damaged mitochondria in destination cells function as “danger” signals.^[57]^ Damaged mitochondria can secrete ROS and cytochrome C, both of which can promote mitochondrial translocation. For instance, cytochrome C produced by damaged mitochondria during the early phases of apoptosis in PC12 cells promotes the production of TNTs, and the use of pancaspase inhibitors prevents the creation of microtubules in TNTs.^[58]^ Mitochondria released from damaged cells can act as damage-associated molecular pattern signals. The capacity of MSCs to transfer and preserve mitochondria from injured cells is improved when MSCs encounter TNTs and are absorbed by another cell because this enhances the production of haemoxygenase 1 and mitochondrial biogenesis.^[59]^

#### 3.1.3. The relationship between mitochondrial transfer and TNTs

TNTs are the main cellular structures that mediate intercellular mitochondrial transfer. Miro1 and Miro2 are connective proteins located in the mitochondrial outer membrane (MOM). In particular, they are well suited for coordinating mitochondrial dynamics with cellular signal control and local energy conversion.^[60]^ In the current model of mitochondrial transfer, miRs on the MOM form a dimer with transport driver binding protein 1, and transport driver binding protein 1 subsequently connects the heavy chain of the mitochondrial motor driver protein kinesin to create a motion adaptation molecular complex that promotes and controls the movement of mitochondria along TNTs. Overexpressing Miro1 in MSCs has been reported to dramatically increase the efficiency of mitochondrial transfer and reverse epithelial damage, whereas inhibiting Miro1 production can result in a reduction in transfer efficiency and repair capacity.^[61]^ Miro1 or Miro2 overexpression or knockout have nearly identical effects on the effectiveness of mitochondrial transport across nerve cells.^[62]^ Target cells can obtain healthy mitochondria via TNTs, while receptor cells can modify their functional properties and escape apoptosis to resume their normal activity.

### 3.2. The roles and mechanisms of mitochondria in the regulation of MSC function

MSCs are pluripotent stem cells that can self-renew and undergo pluripotent differentiation.^[63]^ Additionally, they perform paracrine and immunomodulatory actions and have a wide range of therapeutic applications in cell treatment, functional reconstruction, and regeneration of wounded tissue.^[64]^ The stabilization of the internal environment and control of MSC function depend on cellular morphology, distribution, transfer, biosynthesis, kinetics, and mitochondrial phagocytosis. MSC self-renewal, multidirectional differentiation, senescence, apoptosis, and immunological modulation are all significantly regulated by mitochondria.^[65]^ Additionally, mitochondria can control how MSCs operate by producing ROS, controlling oxidative stress, converting energy metabolism, changing the mitochondrial membrane potential, and other mechanisms.^[66]^ Because mitochondrial oxidative phosphorylation generates a significant quantity of ROS as a byproduct, mitochondria are the primary source of intracellular ROS generation. The activity of MSCs depends heavily on the generation of normal ROS. However, the level of ROS greatly increases under oxidative stress, which might cause major harm to MSCs. Mitochondrial dynamics are the keys to ensuring the optimal morphology of MSCs and enable cells to quickly adapt to environmental pressure, ultimately affecting their self-renewal, differentiation and fate.^[67]^

### 3.3. MSCs and TNTs

MSCs can produce TNTs and use TNTs to deliver mitochondria and other components to target cells.^[68]^ Spees and colleagues initially described mitochondrial transfer from MSCs in 2006. It was noted that A549p cells could not respire or develop aerobically when cocultured with human bone marrow mesenchymal stem cells, but the cocultured cells obtained functioning mitochondria from donor human bone marrow mesenchymal stem cells.^[69]^ Researchers noted the development of TNTs when rat astrocytes or PC12 pheochromocytoma cells were cocultured with MSCs.^[70]^ Studies have shown that infusion of MSCs into ischemic rats can cause effective TNT-mediated mitochondrial transfer, thus promoting the rehabilitation of ischemic stroke.^[71]^ The effects of myocardial ischemia have been shown to be similar. TNT-mediated mitochondrial transfer from MSCs to H9c2 cells guards against ischemia/reperfusion damage in cardiomyocytes.^[72]^ Therefore, mitochondrial transfer from MSCs through TNTs can protect cells by reducing the degree of injury and by supporting the recovery of ischemic tissue.

**Figure 2.**
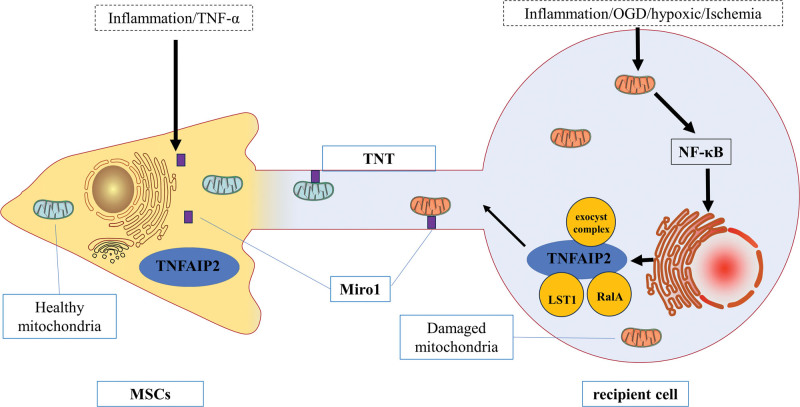
A schematic diagram of the mitochondrial transfer mechanism between MSCs and damaged cells with mitochondrial dysfunction. MSCs = mesenchymal stem cells.

The following mechanisms by which MSCs repair cells have been proposed^[73]^: direct intercellular signal transduction, paracrine signals transmitted by soluble secretory factors (such as hormones and proteins), released exocrine bodies or vesicles containing immunomodulatory molecules and other molecules, and mitochondrial transported by TNTs or microvesicles. Therefore, mitochondrial transfer can rescue many types of injured cells by restoring their bioenergetic needs (Fig. 2).

## 4. The relationship between TNTs and mitochondrial transfer and central nervous system diseases

In ischemic injury, the absence of blood flow causes neurons to suffer irreparable damage, and the neural network no longer functions. Diverse cells, including neurons, astrocytes, microglia, oligodendrocytes, pericytes, and endothelial cells, have diverse reactions to excitotoxicity, oxidative stress, and inflammation. In addition, the integrity and function of the blood–brain barrier are key features for the preservation and recovery of nerve tissue, and TNTs are important “neurovascular units.”^[74]^

### 4.1. TNT-mediated mitochondrial translocation prevents apoptosis and malfunction in nucleus pulposus cells

The use of stem cell-based treatments for several disorders is highly promising. MSCs have distinct immunosuppressive traits, self-renewal capacities, and transdifferentiation capacities. Bone marrow mesenchymal stem cells (BMSCs) can be used as possible therapies. BMSCs can be obtained from adipose tissue and other sources, and many clinical investigations have demonstrated the beneficial effects of MSCs.^[75]^ Research on tissue engineering based on MSCs is the focus of research on the regeneration of intervertebral discs. Nucleus pulposus cells (NPCs) in degenerative intervertebral discs are vulnerable to mitochondrial dysfunction and degeneration. TNT-mediated mitochondrial translocation from BMSCs to NPCs may be a creative way to address this problem. Increased ROS generation spurred by mitochondrial dysfunction causes oxidative damage. Both NPC apoptosis and intervertebral disc degeneration can be caused by mitochondrial dysfunction. The transport network between cells, or TNTs, can transport anything from ions to a whole organelle. TNT-mediated mitochondrial transfer improves the internal balance of recipient cells. Researchers have shown that BMSCs and NPCs govern intercellular mitochondrial transport and produce TNTs in a laboratory setting.^[76]^ To repair tissue damage, a variety of cells, including endothelial cells, cardiomyocytes, and epithelial cells, obtain mitochondria from BMSCs. By transferring mitochondria to recipient cells through TNTs, BMSCs can rescue recipient cells.^[68]^ TNTs effectively mediate intercellular mitochondrial transfer between BMSCs and NPCs, protecting NPCs from mitochondrial dysfunction and death. The repair of the mitochondrial respiratory chain, a rise in the mitochondrial membrane potential, a decrease in the quantity of ROS, and an increase in the rate of apoptosis are all indicators of apoptosis. The key protein governing mitochondrial mobility, Miro1, is not expressed in BMSCs and drastically decreases mitochondrial transfer from BMSCs to NPCs. The mitochondrial adaptation protein Miro1 is responsible for regulating mitochondrial movement.^[77]^ Miro1 is thus a potential target for modulating BMSCs.

In conclusion, this report demonstrated the critical involvement of Miro1 in TNT-mediated mitochondrial transfer from BMSCs and protection of NPCs from mitochondrial dysfunction. These results offer a precise framework for investigating the crucial function of cell–cell contact in intervertebral disc regeneration.

### 4.2. The role of mitochondrial transfer through TNTs in the structural and functional integrity of the blood–brain barrier

To maintain stable neuronal activity, the blood–brain barrier must shield the brain from the constantly changing peripheral environment. Brain endothelial cells, pericytes, microglia, and astrocytes regulate blood–brain barrier exchange, interact with one another, and work together to create the survival signals required to preserve the integrity of the blood–brain barrier and the homeostasis of the central nervous system.^[78]^ Under some pathological conditions that affect the integrity of the blood–brain barrier, such as ischemic stroke, the function of the blood–brain barrier is affected. Moreover, ischemia can also induce mitochondrial morphological changes and apoptosis in astrocytes, resulting in destruction of the blood–brain barrier.^[79]^

Here, TNTs are the primary method by which nearby astrocytes are supplied with functioning mitochondria by distant endothelial cells or pericytes.^[80]^ Pericytes have been discovered to be novel therapeutic targets for ischemic stroke and are thought to be important resources in blood–brain barrier regenerative medicine.^[81]^ In the process of blood–brain barrier repair, pericytes migrate to the injured site. Mitochondrial transfer mediated by TNTs contributes to the survival of astrocytes and the repair of the blood–brain barrier after ischemia, improves the integrity of the blood–brain barrier, prevents neuronal apoptosis and improves neurological function.^[82]^ Interestingly, the recovery of pericytes is similar to the process of rescuing apoptotic cells by MSCs through TNT-mediated mitochondrial transfer.^[83]^ The results confirmed that the interaction of TNTs with blood–brain barrier cells and the mitochondrial transfer of pericytes mediated by TNTs prevented the apoptosis of astrocytes induced by ischemia/reperfusion and confirmed that pericytes may play a key role in maintaining the structural and functional integrity of the blood–brain barrier.

### 4.3. The role of Miro1 in neuronal injury during mitochondrial transfer

Cerebral ischemia caused by stroke is a leading cause of death and disability. The interruption of blood flow leads to the death of neurons and glial cells in many areas of the brain, resulting in brain damage.^[84]^ In the brain and across the neurological system, mitochondria have special and crucial functions. The electrogenesis of neurons consumes the majority of ATP in the brain, whereas mitochondria produce more than 90% of all cellular ATP via oxidative phosphorylation.^[60]^ Since mitochondrial transport and signal transduction are crucial to the existence and function of these cells due to the highly polarized shape and length of neuronal axons, they are tightly controlled.^[85]^

As previously indicated, mitochondria are organelles that are abundantly transferred through TNTs, and Miro1 is a crucial motor protein adaptor and mitochondrial transport regulator, both of which are crucial for mitochondrial transfer. Following oxidative neuronal injury, MSCs use TNTs to transport healthy mitochondria to injured neurons.^[86]^ In oxidatively injured neurons, the protein expression levels of Miro1 and TNFAIP2 increase. The expression levels of Miro1 and TNFAIP2 were restored when MSCs were cocultured with oxidatively injured neurons. When Miro1 is overexpressed, MSCs more efficiently transport mitochondria to injured nerve cells, improving their survival and healing abilities.^[87]^ In contrast, knockout of Miro1 inhibits the production of TNTs and the rescue effect of MSCs, resulting in axonal mitochondrial transport defects; moreover, mitochondrial motility defects lead to neuronal death.^[88]^ In cells damaged by inflammation, TNT-mediated transfer of healthy mitochondria to damaged cells can restore cell function.^[89]^ Improved neuronal survival and functional recovery after stroke may result from MSCs transferring mitochondria to oxidatively damaged neurons.^[90]^ This is a potentially effective treatment for decreasing neuronal loss following ischemia.

### 4.4. Mitochondrial transfer of melatonin through TNTs to repair damaged hippocampal HT22 cells

One of the primary causes of mortality or disability worldwide is cerebral hypoxia or ischemia. Ischaemia is defined by the inability of oxygen and glucose to sustain cellular oxidative metabolism. By inducing oxidative stress, which results in necrosis and apoptosis, postischaemia-reperfusion exacerbates cell damage.^[91]^ One of the primary causes of cell death is thought to be excessive ROS generation by mitochondria during ischemia and reperfusion, particularly in tissues with high energy requirements, such as the brain.^[92]^ Therefore, the maintenance of mitochondrial function following ischemia–reperfusion damage benefits from a decrease in ROS generation. Mitochondrial transfer is a crucial endogenous method of poststroke healing because it may encourage the external replacement of damaged mitochondria and increase cell survival.^[93]^

The pineal gland secretes melatonin, which is subsequently found in the blood and cerebrospinal fluid. Melatonin serves numerous biological purposes.^[94]^ A potent free radical scavenger with a wide antioxidant range, melatonin (N-acetyl-5-methoxytryptamine), can shield tissue from harm due to ischemia/reperfusion.^[95]^ These protective effects are conveyed by a variety of mechanisms, including reducing the amount of mitochondrial ROS induced by ischemia/reperfusion^[96]^; regulating the expression of proinflammatory and anti-inflammatory cytokines to control inflammation and stop the production of cyclooxygenase and inducible nitric oxide synthase^[97]^; enhancing mitochondrial antioxidant enzymes, promoting the production of ATP and reducing the activation of the internal apoptosis pathway^[98]^; activating sirtuin 1, sirtuin 3 (SIRT3) and peroxisome proliferator-activated receptor gamma coactivator-one alpha (PGC1α), which inhibit the accumulation of ROS in mitochondria by increasing the activity of ROS free radical scavenging enzymes and stabilizing mitochondrial function^[99]^; and modulating cytoskeleton structure and function in different types of cells, which can also shield them from free radical damage and aid in cell survival.^[100]^ Increased levels of oxidative stress and ROS buildup occur during mitochondrial failure. The structures of cardiolipin and the mitochondrial electron transport chain are both altered by oxidative stress.^[101]^ These modifications encourage the opening of mitochondrial permeability transition pores, which eventually leads to cell death through apoptosis and autophagy.^[102]^

Neurons are particularly vulnerable to oxidative stress because of their high concentrations of polyunsaturated lipids, rapid oxygen consumption, and low levels of antioxidants and associated enzymes.^[103]^ Melatonin, a potent antioxidant, lowers ROS levels by directly scavenging free radicals and promoting the activity of antioxidant enzymes, including glutathione peroxidase and superoxide dismutase.^[104]^ Additionally, melatonin increased the expression of SIRT3 and PGC1 to improve their capacity to prevent ROS formation. SIRT3/PGC1 may play a role in the protective effect of melatonin, as evidenced by the increase in SIRT3/PGC1 following melatonin administration.^[105]^

**Figure 3.**
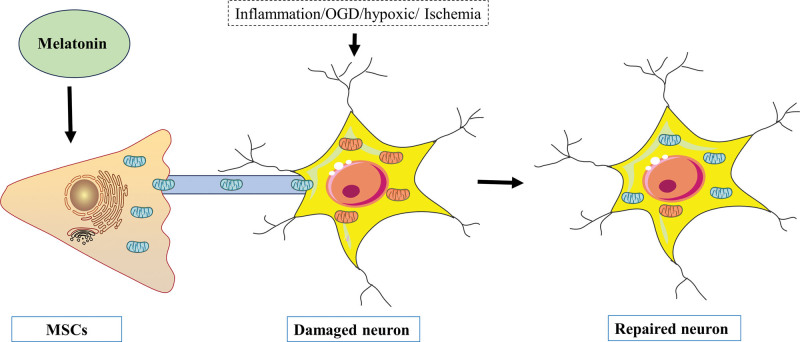
The mechanism of MSCs transporting mitochondria to neurons through TNT under the action of melatonin. MSCs = mesenchymal stem cells. TNT = tunneling nanotube.

Recently, it was discovered that adding melatonin to the cell growth media of cultured hippocampal neurons (HT22 cells) while they are experiencing OGD can minimize the production of ROS and reverse the negative impacts of OGD-induced mitochondrial dysfunction.^[106]^ Importantly, OGD enhanced the production of TNTs in HT22 cells, while melatonin was also associated with an increase in intercellular TNTs. TNTs move mitochondria from healthy cells to apoptotic cells in the wounded area so that the function of the cells can be recovered.^[107]^ The maintenance of cell survival and the quality control of mitochondrial turnover depend on mitochondrial translocation through TNTs.^[108]^ These findings demonstrated that melatonin can delay senescence, stop cellular aging, and prevent cells from dying by preventing mitochondrial damage in injured cells. After melatonin therapy, mitochondrial translocation through TNTs may play a significant role in neuronal survival (Fig. 3).

Conversely, melatonin can also increase the activity of MSCs, improve the homing ability of MSCs after transplantation, reduce the apoptosis rate of MSCs, induce synergistic effects, and reduce inflammation, apoptosis and oxidation.^[109]^ MSCs strongly promote nerve regeneration and good immune characteristics. In various neonatal brain injury models, MSC transplantation can promote neurogenesis, oligodendrocyte growth, axon remodeling and reconnection to promote nerve function recovery. Mitochondrial dysfunction and excessive ROS generation are the major causes of MSC senescence, and melatonin can protect MSCs from this damage.^[109]^ Studies have shown that melatonin therapy rescues aging MSCs in a model of hind limb ischemia in mice, which is related to the enhancement of mitochondrial phagocytosis and improvement of mitochondrial function.^[110]^ In addition, there are many TNTs between MSCs, which allows for the transfer of many substances, including mitochondria, between senescent cells. Determining how melatonin affects MSCs and how it increases stem cell survival is critically important.

In brief, melatonin can improve the mitochondrial function of hippocampal HT22 cells subjected to OGD, decrease ROS production, prevent mitochondrial dysfunction, and transport damaged HT22 cell mitochondria through TNTs.^[111]^ This is a novel mechanism by which melatonin protects against ischemia/reperfusion injury.

## 5. Future prospects

TNFAIP2 plays an important role in the formation and development of TNTs. TNTs are a novel method of intercellular communication. Mitochondrial transfer through TNTs is an important cellular repair method, and this mechanism may be useful for the treatment of many diseases. Therefore, one of the necessary future research directions is to further improve the maneuverability of mitochondrial transplantation, to understand autologous MSC-mediated mitochondrial transfer and to clarify the mechanisms, signaling targets and modes of action of mitochondrial energy metabolism in MSCs.

## Acknowledgments

We thank American Journal Experts (AJE) for the certification number 5E98-374B-F442-1FC4-FCFP for providing English language editing for our manuscript.

## Author contributions

**Formal analysis:** Ye Chen, Xihong Li, Dongqiong Xiao.

**Investigation:** Ye Chen.

**Supervision:** Dongqiong Xiao.

**Writing – original draft:** Ye Chen.

**Writing – review & editing:** Ye Chen, Dongqiong Xiao.
